# The H Syndrome: A Genodermatosis

**DOI:** 10.7759/cureus.2763

**Published:** 2018-06-08

**Authors:** Shoaib Bhatti, Asma Jamil, Samrah Hasan Siddiqui, Uzair Yaqoob, Luqman Naseer Virk, Areesh Bhatti

**Affiliations:** 1 Peadiatric Medicine, National Institute of Child Health, Karachi, PAK; 2 Paediatric Medicine, National Institute of Child Health, Karachi, PAK; 3 Medicine, Dow University of Health Sciences, Karachi, PAK; 4 Sindh Medical College, Dow University of Health Sciences, Karachi, PAK; 5 Medicine, Aga Khan University, Karachi, PAK

**Keywords:** h syndrome, histiocytosis, lymphadenopathy, autoimmunity

## Abstract

H syndrome (histiocytosis lymph adenopathy plus syndrome) is an autosomal recessive disorder caused by mutations in the SLC29A3 gene, encoding the human equilibrative nucleoside transporter (hENT3), characterized by cutaneous hyperpigmentation and hypertrichosis, hepatosplenomegaly, hearing loss, heart anomalies, hypogonadism, low height, hyperglycemia/insulin-dependent diabetes mellitus, and hallux valgus/flexion contractures. Exophthalmos, malabsorption, renal anomalies, flexion contractions of interphalangeal joints and hallux valgus, and lytic bone lesions, as well as osteosclerosis, are also seen. If these are lacking, the constellation of additional findings should raise suspicion for H syndrome. As most of the patients reported to date with H syndrome are from traditional, low-income populations, where consanguinity is common, it is highly important to develop a cheap and affordable technique for a mutation analysis. Two siblings presented to us, diagnosed as having insulin-dependent diabetes mellitus (IDDM) since the age of eight years and progressive flexion contracture of the small joints for seven-eight years. On examination, both had short stature. One also had bilateral cervical lymphadenopathy. The female had the Tanner stage of B3P3A2 M0 and the male had the Tanner stage of prepuberty. Laboratory workup, including antinuclear antibodies, rheumatoid factor, erythrocyte sedimentation rate, thyroid profile, and Celiac serology were negative. Genetic studies confirmed the diagnosis of H syndrome.

## Introduction

H syndrome (histiocytosis lymph adenopathy plus syndrome) is an autosomal recessive syndrome, which is caused by mutations in the SLC29A3 gene, encoding the human equilibrative nucleoside transporter (hENT3) [1]. H syndrome is a multisystemic disorder characterized by cutaneous hyperpigmentation, hypertrichosis, hepatosplenomegaly, hearing loss, heart anomalies, hypogonadism, low height (short stature), hyperglycemia/insulin-dependent diabetes mellitus (IDDM), and hallux valgus/flexion contractures [2-3]. 

## Case presentation

We describe two siblings from a Pakistani consanguineous family, 18-year-old female (ABC) and 15-year-old male (XYZ) diagnosed as having IDDM since the age of eight years, with progressive flexion contracture of the small joints of both hands and feet for seven to eight years. One female child of the family expired at the age of eight years with a similar complaint of IDDM and joint contracture.

**Figure 1:**
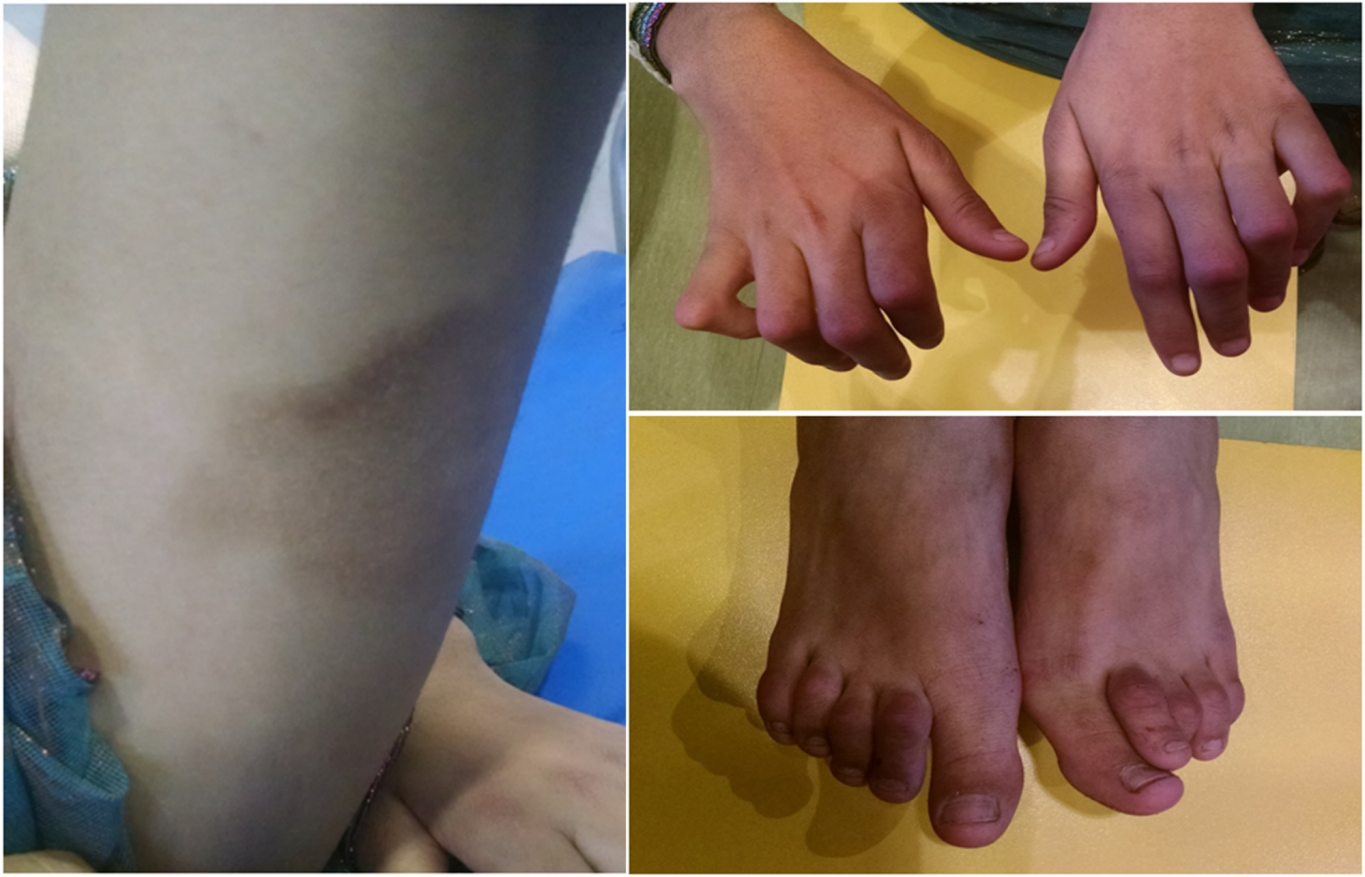
Dermatological manifestations and limb appearance of female child

On examination, ABC had short stature with standard deviations (SDS) of -2.48 and a hyperpigmented skin lesion at the posterior medial aspect of both thighs without hypertrichosis. She had a Tanner stage of B3P3A2 M0. She also had a contracture of the proximal metacarpophalangeal joint of both hands and a contracture with plantar flexion of the metatarsophalangeal joints of the bilateral feet with restriction of both active and passive movement (Figure 1).

**Figure 2:**
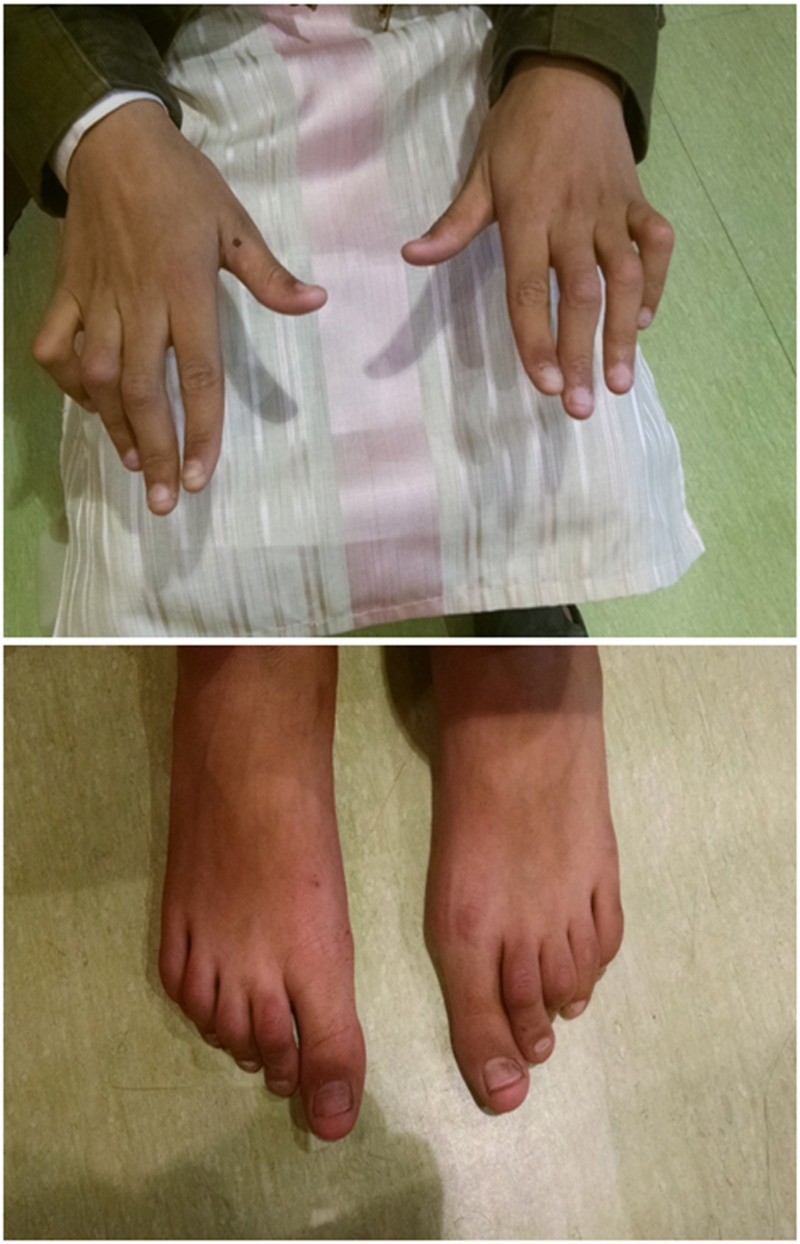
Limb manifestations of male child

On examination, XYZ also had short stature with SDS of -3.3. He had bilateral cervical lymphadenopathy and a Tanner stage of prepuberty. He had a similar contracture of the proximal metacarpophalangeal joints of both hands and a contracture with plantar flexion of the metatarsophalangeal joints of bilateral feet with restriction of both active and passive movement (Figure 2).

In both siblings, there were no signs of inflammation and no large joint involvement. The laboratory workup of both siblings, including antinuclear antibodies (ANA), rheumatoid factor (RA factor), erythrocyte sedimentation rate (ESR), thyroid profile, and Celiac serology, were negative. A genetic study for monogenic diabetes revealed a homozygous nonsense mutation in the SCL29A3 gene in both. A genetic study of both parents revealed a heterozygous mutation in the SCL29A3 gene. This result confirmed a genetic diagnosis of histiocytosis lymph adenopathy plus syndrome (also known as H syndrome). The patient left against medical advice before further workup and treatment.

## Discussion

H syndrome is a rare, systemic, inherited histiocytosis with a prevalence of < 1/1000. To date, around 100 patients have been described in the world literature. H syndrome becomes clinically apparent during childhood, and cutaneous features are the most prevalent [4]. Sensorineural hearing loss is the second most common manifestation. Additional features include heart anomalies, hepatosplenomegaly, lymphadenopathy, insulin-dependent diabetes mellitus, hypogonadism, angiopathy, and genital masses. Exophthalmos (with normal thyroid function), malabsorption (due to pancreatic exocrine insufficiency), renal anomalies, flexion contractions of the interphalangeal joints and hallux valgus, and lytic bone lesions, as well as osteosclerosis, are also seen. Diagnosis is suspected by the pathognomonic cutaneous features. If these are lacking, the constellation of additional findings should raise suspicion for H syndrome. Diagnosis is confirmed by genetic screening for SLC29. Management is mainly supportive. Oral steroids may temporarily improve cutaneous changes. Early screening for sensorineural hearing loss and diabetes mellitus should be performed [4].

## Conclusions

As most of the patients reported to date with H syndrome are from traditional, low-income populations, where consanguinity is common, it is highly important to develop a cheap and affordable technique for mutation analysis. Endocrine manifestations are a major constituent of H syndrome, but it is hardly reported in the endocrine literature published to date. Therefore, this case report is a very welcome contribution, as it emphasizes the importance of recognizing H syndrome among endocrinologists. IDDM, hypogonadism, and short stature, in association with cutaneous hyperpigmentation, hypertrichosis, hearing loss, or unexplained phalangeal contractures should alert endocrinologists to evaluate the possibility of H syndrome.
